# Rapid atrial pacing above the maximum sensor rate: a case report

**DOI:** 10.1093/ehjcr/ytad586

**Published:** 2023-11-21

**Authors:** George M Bodziock, Patrick M Kozak, Carrie Pruitt, John F Dillon, Prashant D Bhave

**Affiliations:** Section of Cardiovascular Medicine, Cardiac Electrophysiology, Wake Forest University School of Medicine, 1 Medical Center Blvd, Winston-Salem, NC 27157, USA; Section of Cardiovascular Medicine, Cardiac Electrophysiology, Wake Forest University School of Medicine, 1 Medical Center Blvd, Winston-Salem, NC 27157, USA; Section of Cardiovascular Medicine, W.G. Hefner Salisbury Department of Veterans Affairs Medical Center, K1601 Brenner Ave, Salisbury, NC 28144, USA; Section of Cardiovascular Medicine, Cardiac Electrophysiology, Wake Forest University School of Medicine, 1 Medical Center Blvd, Winston-Salem, NC 27157, USA; Section of Cardiovascular Medicine, Cardiac Electrophysiology, Wake Forest University School of Medicine, 1 Medical Center Blvd, Winston-Salem, NC 27157, USA

**Keywords:** Case report, Pacemaker, Atrial pacing, Rapid pacing, Programming

## Abstract

**Background:**

While ventricular-based timing modes are known to cause elevated atrial pacing above the lower rate when intrinsic atrioventricular (AV) conduction is shorter than programmed AV delay, there is one case report in 2015 by Jafri *et al.* where rapid atrial pacing was induced in an Abbott device set DDI with a lower rate of 90 by an unsensed premature atrial complex and slow intrinsic AV conduction allowing pacemaker ‘crossover.’

**Case summary:**

We present a very unusual case of rapid atrial pacing at >180 b.p.m. due to a perfect storm of events that we believe has not been previously reported. A patient with a St. Jude Abbott DCPPM set DDDR had an atrial tachyarrhythmia causing a mode switch to DDIR, which uses ventricular-based timing. This was followed by a period of rapid atrial pacing that terminated spontaneously.

**Discussion:**

This phenomenon depended on an initial atrial tachyarrhythmia causing a mode switch to DDIR. In addition, the set lower rate would not have led to a short enough calculated ventriculo-atrial interval (VAI), but because rate responsive pacing was enabled, the calculated VAI was short enough to promote the crossover in setting of slow AV conduction and allow the rapid atrial pacing. Understanding this unique mechanism requires careful attention to pacemaker timing cycles and appreciation of the limitations of device programming. While it appears that a similar phenomenon was reported once in the literature, we believe that this episode of rapid atrial pacing was even more serendipitous due to the unlikely series of events required for its inception.

**Learning Points**

- While modern pacemakers predominantly use atrial-based timing, some companies still use ventricular-based timing for non-tracking modes such as DDI or DDIR.
- Rapid atrial pacing in ventricular-based timing mode, above the lower rate or max sensor indicated rate, can be seen when intrinsic atrioventricular (AV) conduction is shorter than programmed AV delay.
- Rarely after a perfectly timed premature atrial complex in ventricular-based timing mode, rapid atrial pacing may also be seen when intrinsic AV conduction is prolonged and there is pacemaker ‘crossover’ with short ‘pseudo’ AV conduction.

## Introduction

Modern dual-chamber pacemakers (DCPPM) typically operate in atrial-based timing when programmed DDD, where the atrial–atrial (A–A) interval is fixed and the ventriculo-atrial interval (VAI, also known as the atrial escape interval) varies.^1^ However, when certain devices are set to DDI or automatic mode switch (AMS) to non-tracking DDI programming, pacing rate is determined by ventricular-based timing where the calculated VAI is fixed and the A–A can vary.^1^ In this case, VAI is calculated based on the base rate and the programmed paced atrioventricular (AV) delay.^1^ For example, if the set lower rate is 60 b.p.m. (1000 ms) with paced AV delay set at 250 ms, the calculated VAI would be equal to 750 ms (1000 − 250 = 750). When native AV conduction is shorter than programmed AV delay, the VAI will remain the same but the A–A will shorten. This can lead to atrial pacing above the lower rate. For the previous example, if native AV conduction was 120 ms, the calculated VAI would remain 750 ms, and the effective atrial pacing rate would be 120 + 750 = 870 ms (68 b.p.m.). While this phenomenon is well described, there is a lesser known scenario where prolonged intrinsic AV conduction can also lead to atrial pacing above the set rate.^2^

## Summary figure

**Figure:**
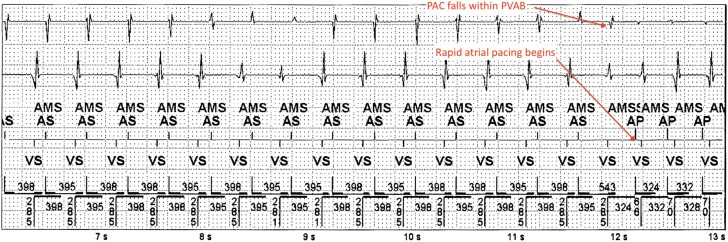


## Case report

**Figure 1:**
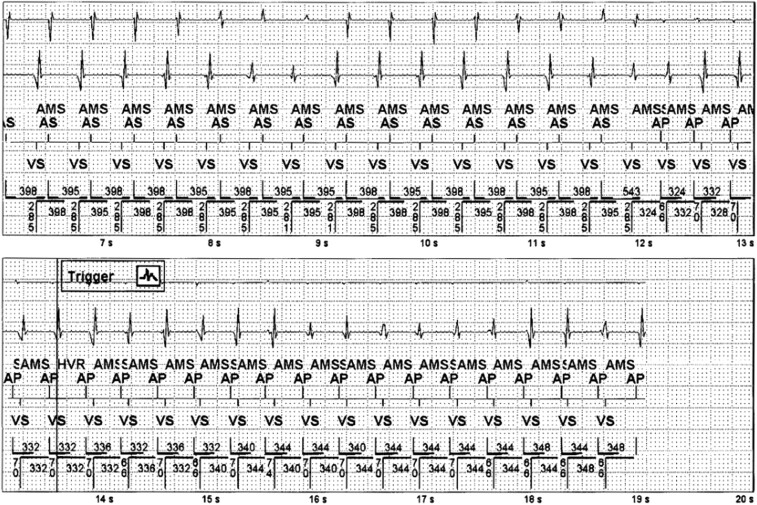
EGM with AT and prolonged AV conduction. Automatic mode switch is triggered setting device DDIR. There is PAC in the post-ventricular atrial blanking period (PVAB) followed by rapid atrial pacing at 330–340 ms (180 b.p.m.).

A 74-year-old man with sick sinus syndrome and a St. Jude Abbott DCPPM, placed 8 months prior, presented for routine device check. Presenting rhythm was atrial paced (AP) ventricular sensed (VS) at 70 b.p.m. Atrial and ventricular impedance, sensing, and thresholds were appropriate and consistent with prior values. Device settings were DDDR with base rate 70 b.p.m., maximum tracking rate 105 b.p.m., and maximum sensor rate 130 b.p.m., and paced and sensed AV delays were 250 ms. Ventricular intrinsic preference was enabled, allowing intermittent AV delay to promote intrinsic conduction and mitigate ventricular pacing. A ventricular high rate episode with corresponding electrogram (EGM) was noted (*Figure 1*). This episode commenced with a 1:1 atrial tachycardia (AT) at a rate of 150 b.p.m. with prolonged AV conduction, prompting AMS from DDDR to DDIR. Unexpectedly, this was followed by a period of rapid atrial pacing at 180–190 b.p.m., well exceeding the maximum tracking and sensor rates. Notably, there was no atrial anti-tachycardia pacing programmed on this device, and the native AV conduction was longer than the programmed AV delay. In addition, the observed AV interval during rapid atrial pacing appeared implausibly short. To understand the mechanism for this phenomenon, one must consider how pacemaker timing cycle parameters determine the pacing rate.

**Figure 2:**
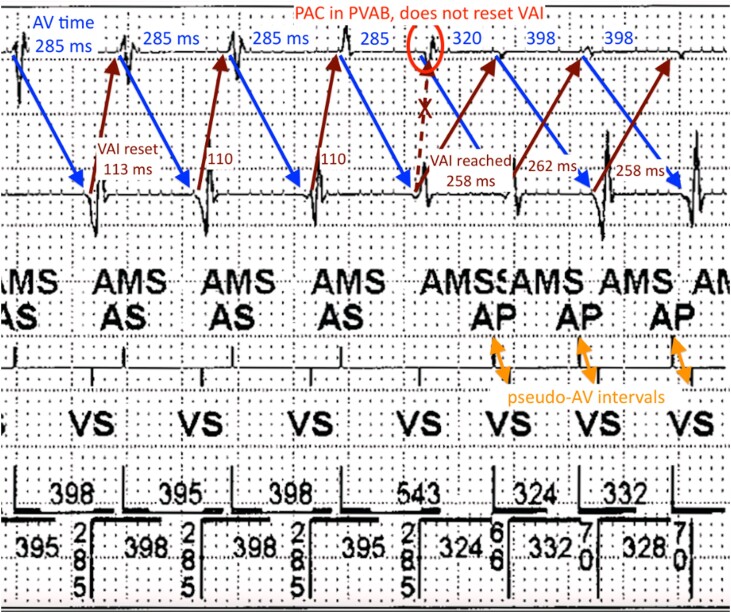
Blue downward arrows mark conducted AV intervals. Dark red upward arrows show VAI (also known as the atrial escape interval) that is reset by each AS event. An unsensed PAC (red circle) falling within PVAB (no label or marker) delays the next beat of AT until VAI times out, triggering AP. Subsequent APVS crossover occurs with short pseudo-AV interval (orange double sided arrows).

In ventricular-based timing modes, the cycle length between two atrial-paced events is determined by the calculated VAI, not the A–A interval as in atrial-based timing.^1^ This calculation depends on the base rate, or sensor indicated rate, and the programmed paced AV delay.^1^ This patient’s device was initially set to DDDR. While in an episode of AT, the device mode switched from atrial-based timing mode to a ventricular-based timing mode (DDIR). As shown in *Figure 2*, the initial AT cycle length was ∼395 ms triggering AMS to DDIR, and intrinsic AV time was prolonged at 285 ms. Then, a perfectly timed premature atrial complex (PAC) fell within the post-ventricular atrial blanking period (PVAB) and was not sensed, thus failing to reset the VAI timer. With further AV nodal decrement, this PAC conducted with an AV time of 320 ms. However, the calculated VAI (atrial escape interval) had already elapsed at 258 ms, leading to an AP beat just before the PAC conducted to a ventricular-sensed event. This ‘pacemaker crossover’ created a short ‘pseudo-AV interval’ of 66 ms, and the ventricular-sensed event from the conducted PAC resets the VAI timer just after the AP beat. Ventriculo-atrial interval was then reached again at ∼260 ms, triggering another AP event with even slower AV time of 398 ms, once again ‘crossing over’ with AP occurring before VS event from the preceding paced beat. This consistent but prolonged AV conduction led to repetitive crossover with short pseudo-AV intervals resetting VAI. The VAI was calculated based on sensed activity, due to enabled rate responsive pacing, and an assumed paced AV time of 250 ms, leading to atrial pacing well above the max sensor rate.

This event was stored as a ventricular high rate episode because the ventricular rate exceeded 175 b.p.m.; however, it lasted <20 s, and the stored EGM did not capture the termination. Presumably, it terminated with an atrial-paced beat that failed to conduct, or due to a spontaneous PAC or pemature ventricular contraction. Importantly, there were no associated symptoms with this brief episode, nor were there any other similar episodes recorded before or within 6 months following. To prevent this episode from happening again, multiple programming changes could be made including changing the AMS from DDIR to a non-sensor setting such as DDI or VVI; reducing the maximum sensor driven rate, or shortening the programmed AV delay. However, each of these changes would have other implications. Therefore, another option would be to make no programming changes given that this was a single short episode with no associated symptoms. Even if lightning did strike twice, these episodes would be unlikely to sustain for any significant length of time given reliance on extremely long yet necessarily fixed 1:1 AV conduction. In the case of this patient, the company representative was contacted and agreed with our proposed mechanism and potential programming changes to prevent recurrence. However, ultimately no changes were made, and the phenomenon has not recurred since this initial episode.

## Discussion

Jafri *et al.*^2^ reported a unique case of rapid atrial pacing at 150 b.p.m. in an Abbott device set DDI with lower rate of 90 b.p.m., which started with a series of PACs that fell just outside PVAB of the preceding AP event; and with prolonged AV time, led to pacing ‘crossover’ with delayed VS event occurring just after the next AP event. In our case, a similar mechanism was the culprit, but was even more serendipitous. The mechanism depended on transitioning from DDD to DDI mode during an atrial tachyarrhythmia, a perfectly timed PAC, prolonged intrinsic AV conduction, and rate responsive pacing. Since calculated VAI was ∼260 ms with a programmed AV time of 250 ms, the intended sensor driven rate (A–A) would have been equal to VAI + AV = 510 ms, meaning that the device was calculating VAI with the intent of effective atrial pacing at 510 ms (118 b.p.m.). This elevated pacing rate, in the setting of prolonged intrinsic AV conduction, led to the ‘crossover’ that generated rapid pacing. In DDI mode without rate responsive pacing, the device would have calculated VAI with intent to pace at the set lower rate of 70 b.p.m. (857 ms) that would have yielded a calculated VAI of 607 ms, which would have given ample time for the initial PAC to conduct before VAI was reached. In fact, any calculated VAI longer than 320 ms (the intrinsic AV conduction after the PAC) would have prevented crossover and therefore prevented rapid atrial pacing. This means that the episode could only happen with enabled rate responsive pacing for a target heart rate > 105 b.p.m.

## Conclusion

With ventricular-based pacing modes, atrial pacing at rates surpassing the lower set rate is a known phenomenon when intrinsic AV conduction is shorter than programmed paced AV delay.^1^ While Jafri *et al.* previously reported a case of elevated atrial pacing in the setting of prolonged AV conduction, we report a unique case with similar mechanism, but which also required an atrial tachyarrhythmia causing a mode switch, as well as rate adaptive pacing. This series of events has not been previously reported, requiring a perfect storm of tachyarrhythmia-induced AMS to ventricular-based timing, a precisely timed single fortuitous PAC, sustained slow pathway conduction, and an elevated sensor driven rate exceeding 105 b.p.m.

## Data Availability

All data and findings to support this case report are included in the article.

## Lead author biography

After completing medical school at Dartmouth in 2016, I completed internal medicine residency at Vanderbilt in 2019 and general cardiology residency at Wake Forest in 2022. I am currently a rising second year electrocardiology fellow at Wake Forest University School of Medicine.

**Consent:** Informed consent for patient information and images to be published was provided by the patients in accordance with COPE guidelines.

**Funding:** None declared.
